# Supplementary material for: The implications of alternative splicing regulation for maximum lifespan
Source: Nat Commun. 2025 Nov 24;16:10317. doi: 10.1038/s41467-025-65339-1 (PMC12644568; doi:10.1038/s41467-025-65339-1)
Supplement: Supplementary file 1 — Supplementary Information [file 41467_2025_65339_MOESM1_ESM.pdf]

Supplementary Fig. 1. Workflow of alternative splicing identification and quantification. (a) nonhuman mammal species. (b) humans. *For mouse samples, gene annotations from GENCODE (Mus_musculus.GRCm39.107) were used for transcript quantification instead of de novo transcriptome assembly.

Supplementary Fig. 2. Sequence regions used for identification of homologous alternative splicing. (a) exon skipping (cassette exons); (b) mutually exclusive exons; (c) alternative 5' splice sites (alternative donors); (d) alternative 3' splice sites (alternative acceptors); (e) intron retention; (f) alternative first exons; (g) alternative last exons.

Supplementary Fig. 3. Seven types of alternative splicing events. (a) AS events within individual species. (b) Conserved AS events with homologs (against mice) in at least 10 species.

Supplementary Fig. 4. Type distribution of MLS-associated alternative splicing events

Supplementary Fig. 5. Distribution and Q-Q plots of gene expression levels for genes with splicing events positively (Pos) or negatively (Neg) correlated with maximum lifespan in six tissues.

Supplementary Fig. 6. Percentage of MLS-associated alternative splicing (AS) events with significant PSI increases across varying ΔPSI thresholds. Significance of the PSI increase was determined using a one-sided Fisher's exact test on median read counts, with the false discovery rate (FDR) controlled at < 0.05 using the Benjamini-Hochberg method. Lines represent MLS positively correlated, MLS negatively correlated, and background event groups.

Supplementary Fig. 7. Type distribution of age-MLS overlapping alternative splicing events.

Supplementary Fig. 8. Heatmap showing hierarchical biclustering of age-associated AS events based on RBP motif enrichment. Each row corresponds to a specific AS event, while columns represent the associated RBPs.

Supplementary Fig. 9. Heatmap of pairwise tissue similarity for age-associated splicing events that are identified by traditional regression models fitting alternative splicing individually. The similarity score is based on Jaccard index values.

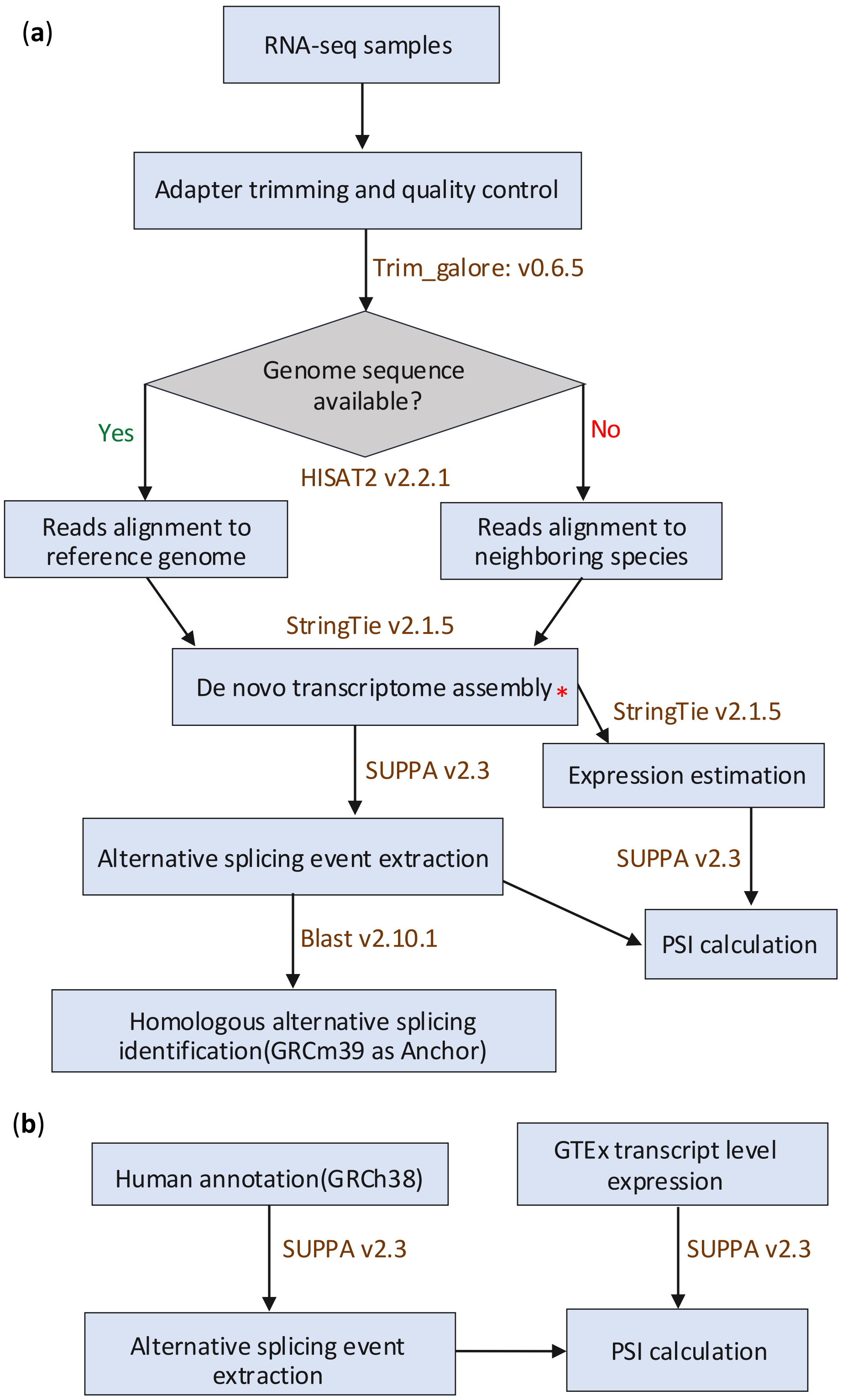

Supplementary Fig. 1. Workflow of alternative splicing identification and quantification. (a) nonhuman mammal species. **(b)** humans. *For mouse samples, gene annotations from GENCODE (Mus_musculus.GRCm39.107) were used for transcript quantification instead of de novo transcriptome assembly.

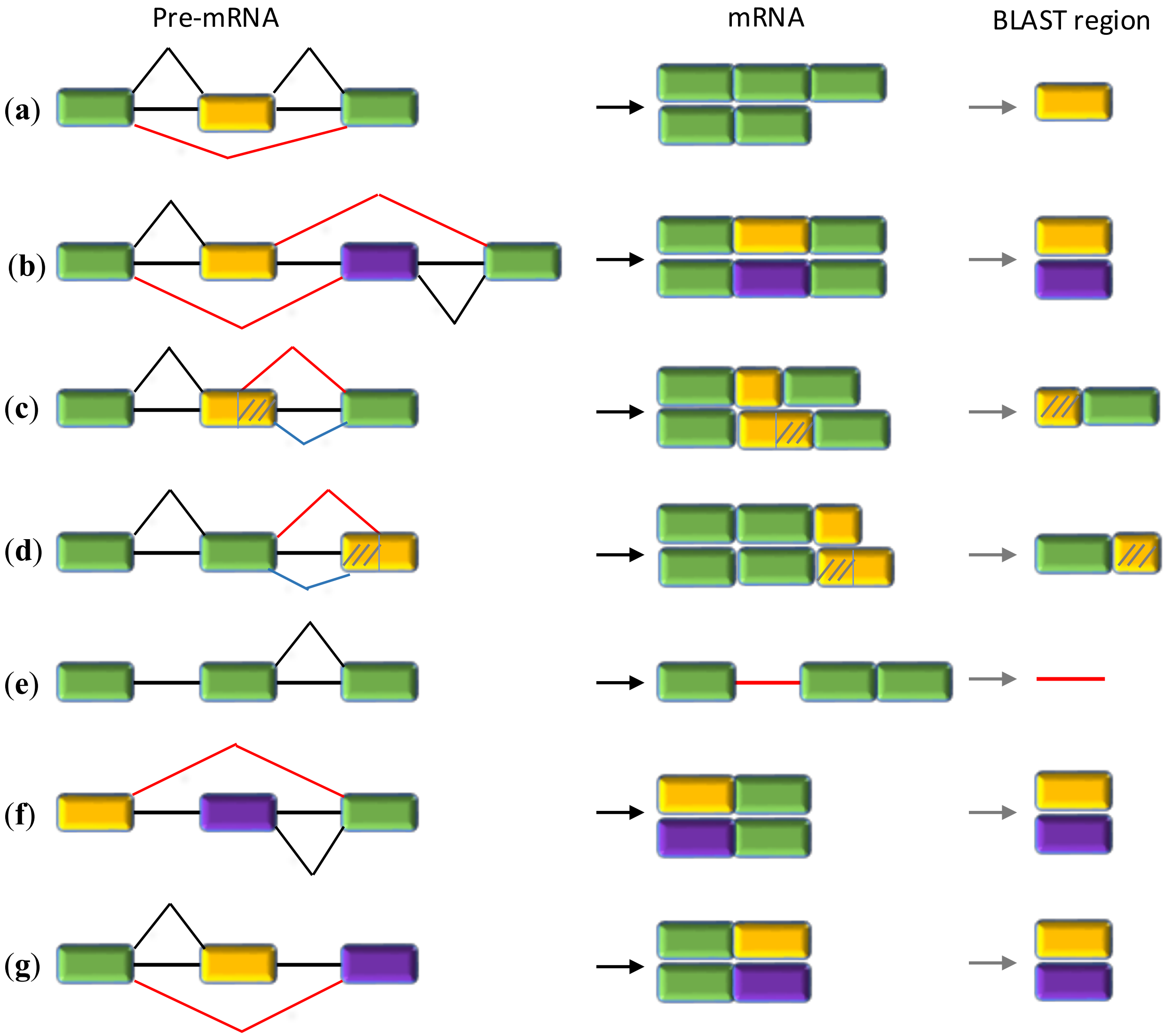

Supplementary Fig. 2. Sequence regions used for identification of homologous alternative splicing. (**a**) exon skipping (cassette exons); (**b**) mutually exclusive exons; (**c**) alternative 5’ splice sites (alternative donors); (**d**) alternative 3’ splice sites (alternative acceptors); (**e**) intron retention; (**f**) alternative first exons; (**g**) alternative last exons.

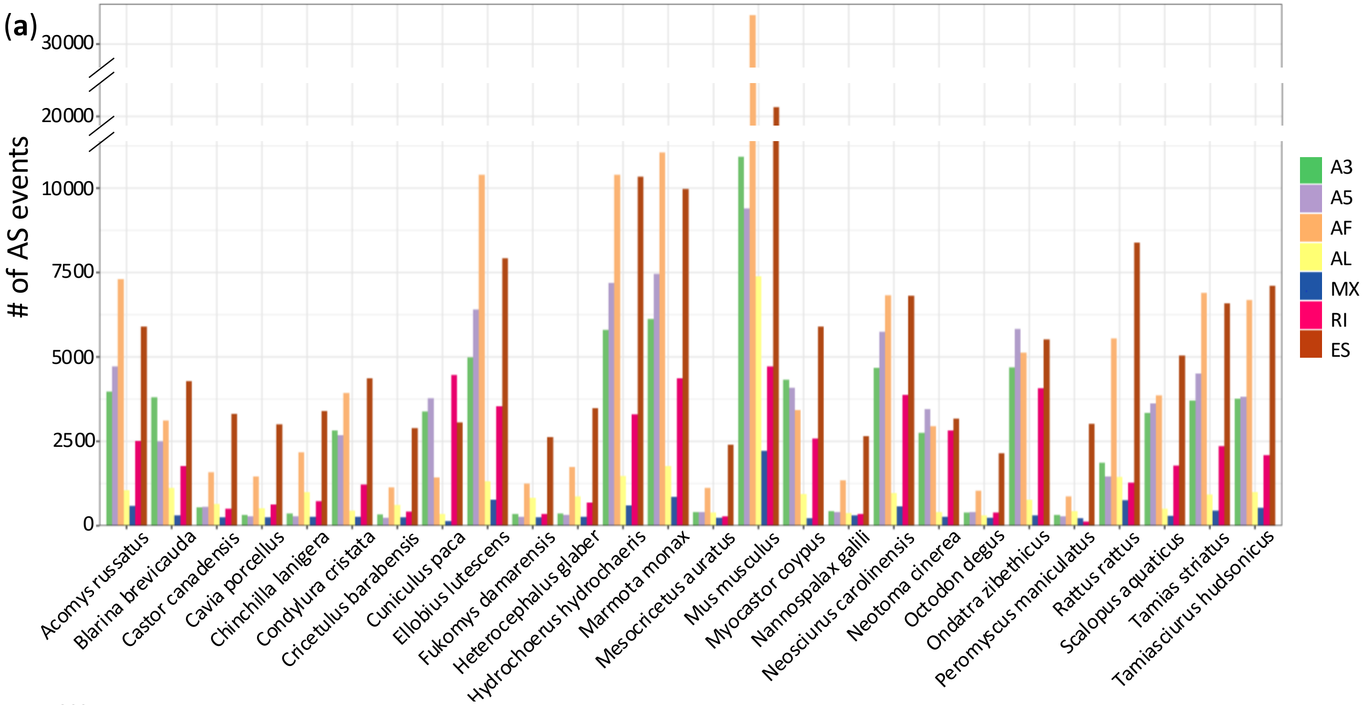

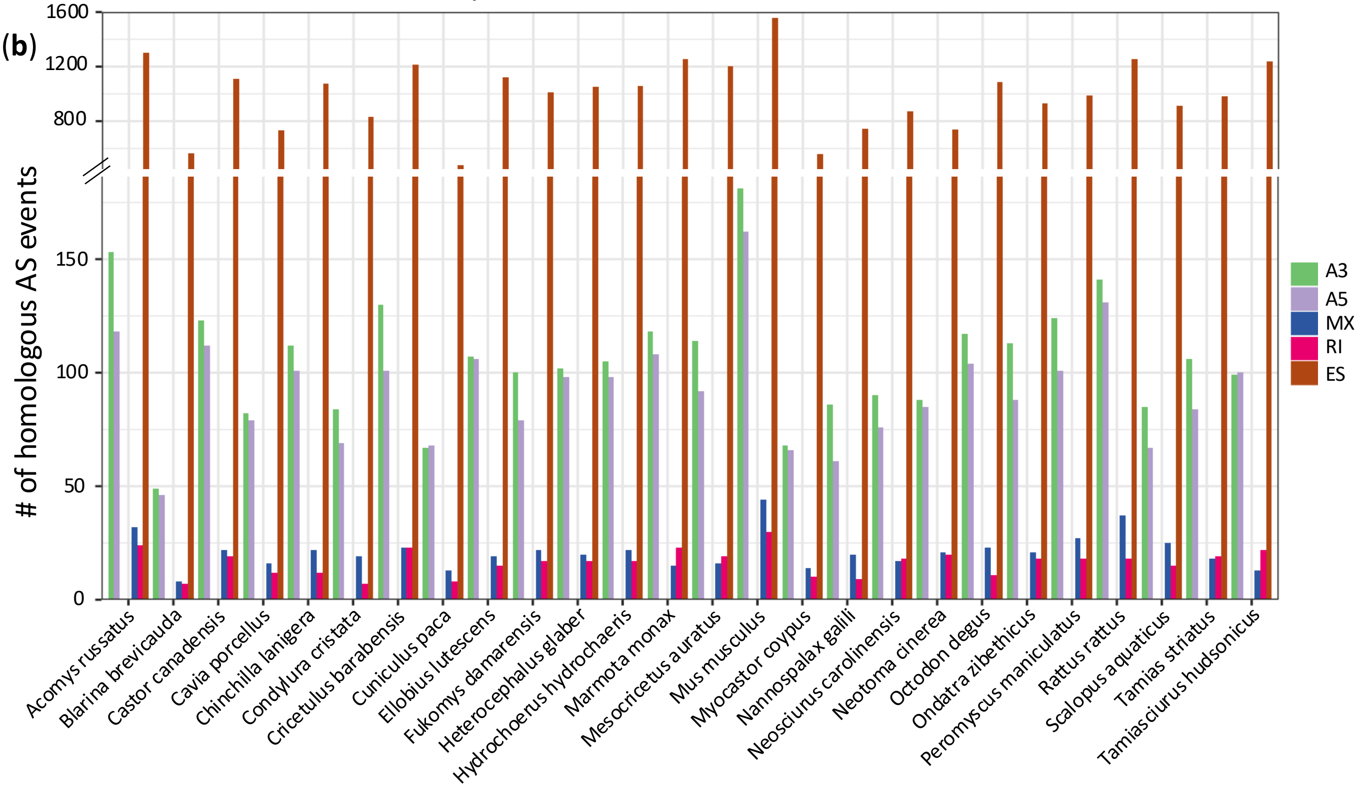

Supplementary Fig. 3. Seven types of alternative splicing events. (**a**) AS events within individual species. (**b**) Conserved AS events with homologs (against mice) in at least 10 species.

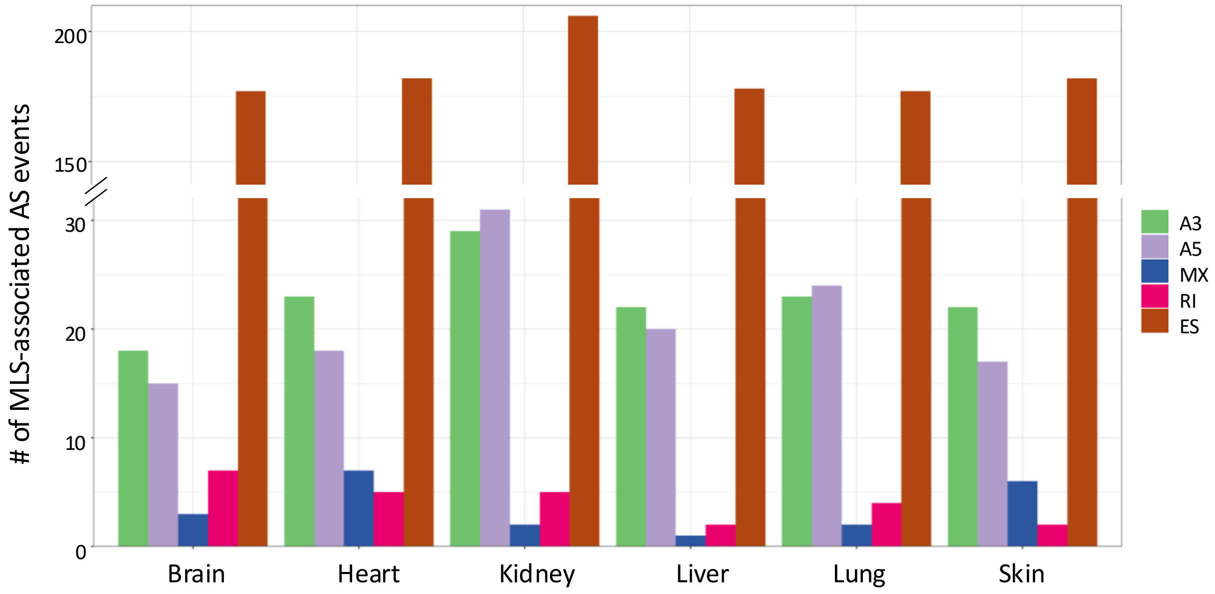

Supplementary Fig. 4. **Type distribution of MLS-associated alternative splicing events.**

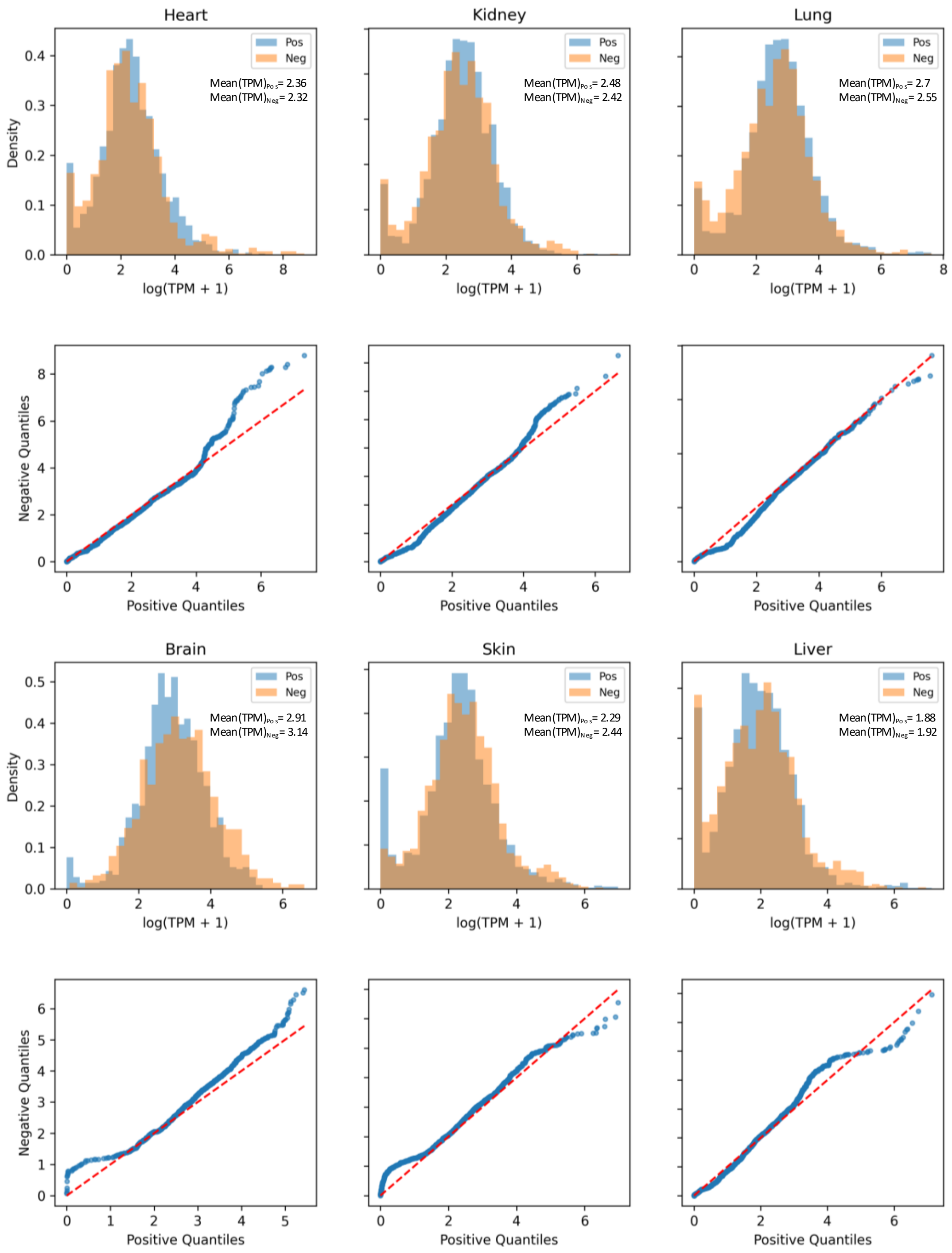

Supplementary Fig. 5. Distribution and Q-Q plots of gene expression levels for genes with splicing events positively (Pos) or negatively (Neg) correlated with maximum lifespan in six tissues.

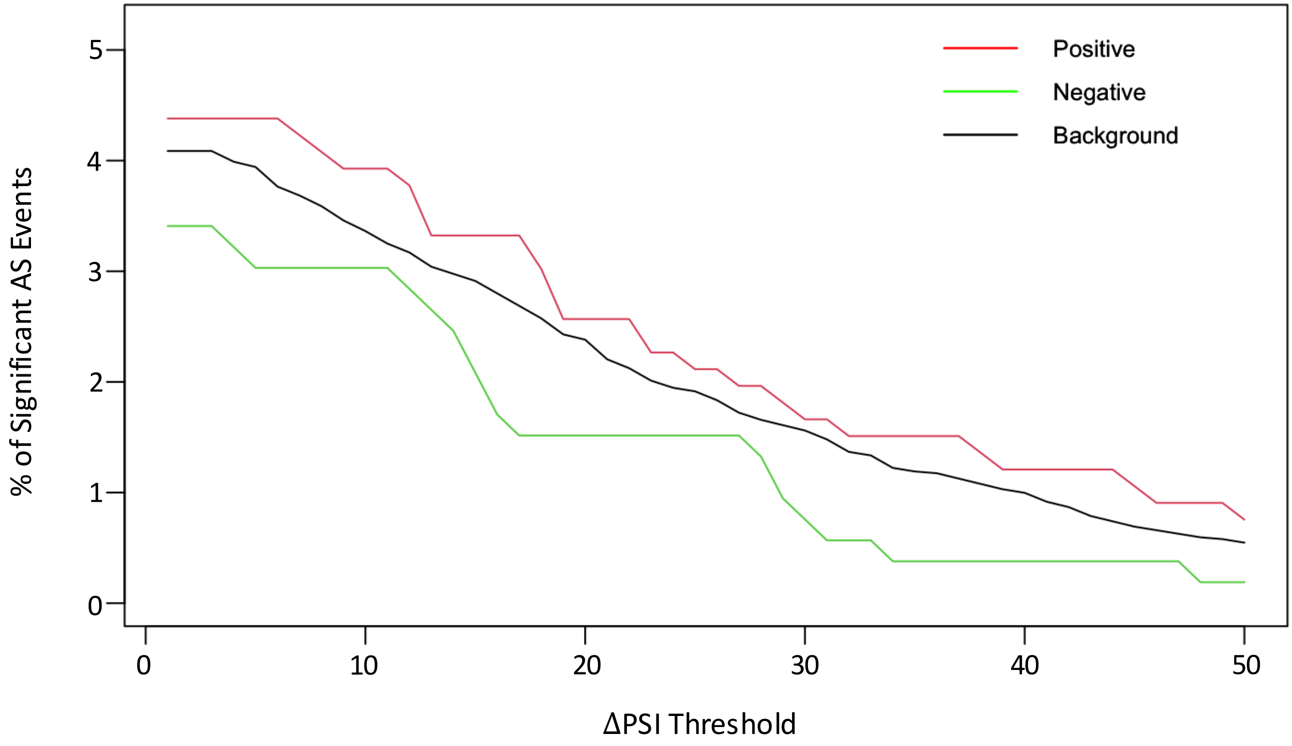

Supplementary Fig. 6. Percentage of MLS-associated alternative splicing (AS) events with significant PSI increases across varying ΔPSI thresholds. Significance of the PSI increase was determined using a one-sided Fisher's exact test on median read counts, with the false discovery rate (FDR) controlled at < 0.05 using the Benjamini-Hochberg method. Lines represent MLS positively correlated, MLS negatively correlated, and background event groups.

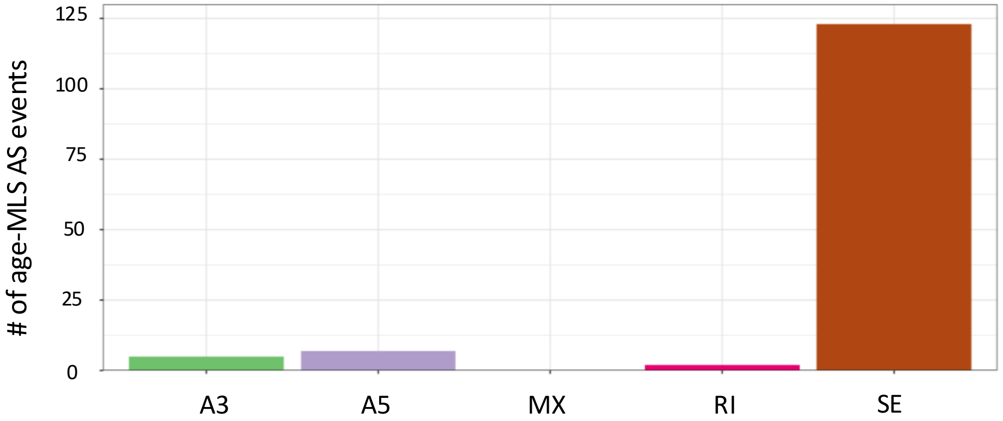

Supplementary Fig. 7. **Type distribution of age-MLS overlapping alternative splicing events.**

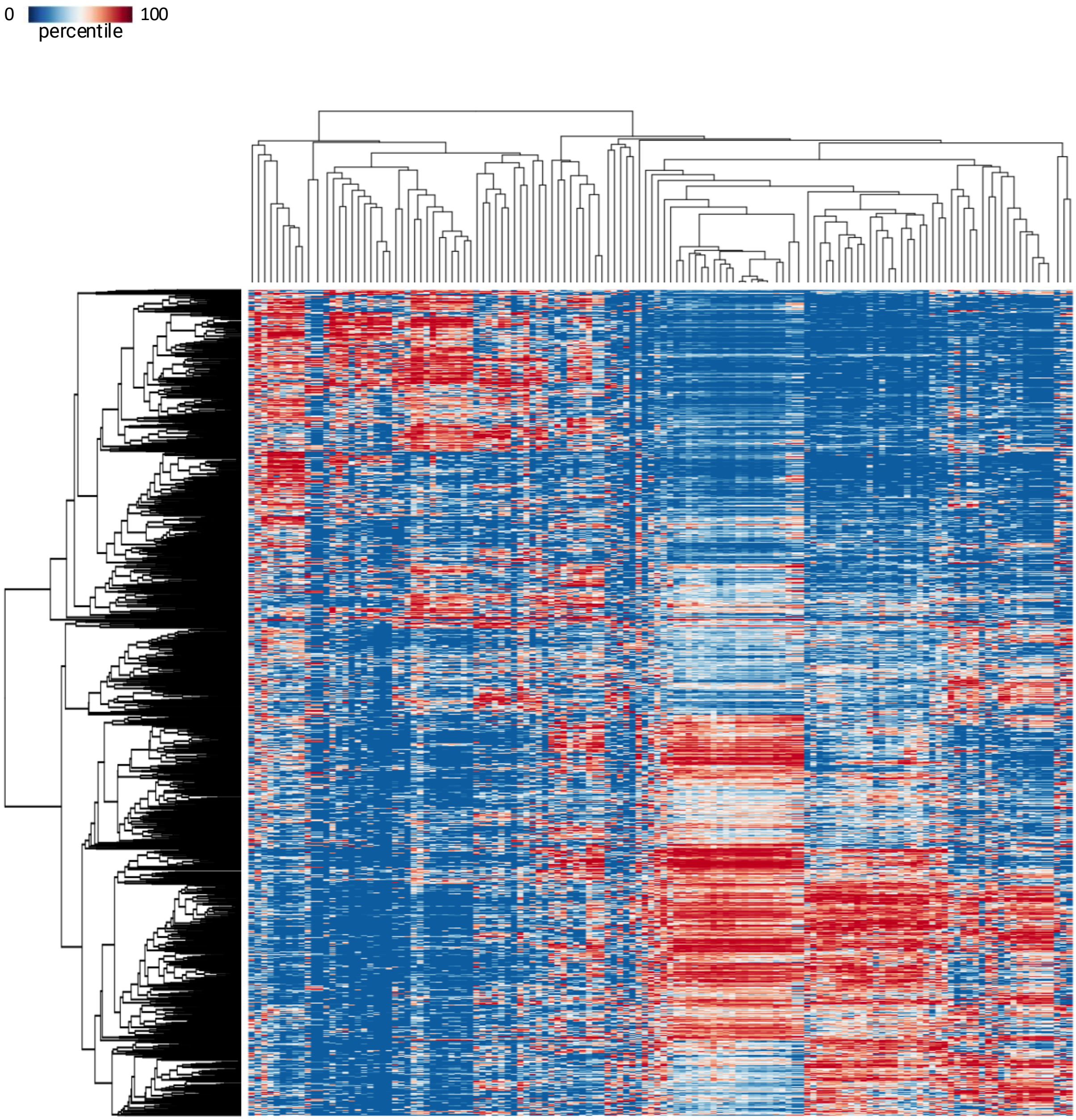

Supplementary Fig. 8. Heatmap showing hierarchical biclustering of age-associated AS events based on RBP motif enrichment. Each row corresponds to a specific AS event, while columns represent the associated RBPs.

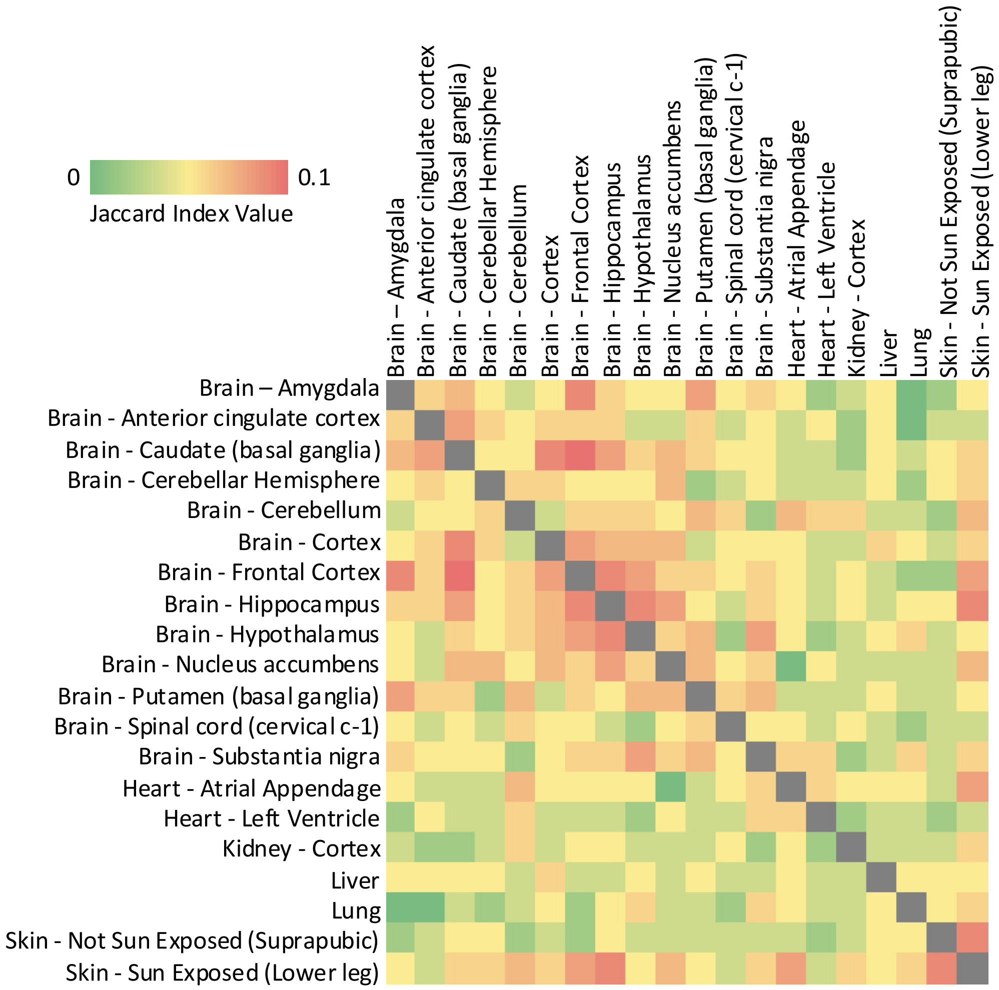

Supplementary Fig. 9. Heatmap of pairwise tissue similarity for age-associated **splicing events that are identified by traditional regression models fitting alternative splicing individually.** The similarity score is based on Jaccard index values.
