# Supplementary material for: The implications of alternative splicing regulation for maximum lifespan
Source: Nat Commun. 2025 Nov 24;16:10317. doi: 10.1038/s41467-025-65339-1 (PMC12644568; doi:10.1038/s41467-025-65339-1)
Supplement: Supplementary file 2 — Description of Additional Supplementary Files [file 41467_2025_65339_MOESM2_ESM.pdf]

Description of Additional Supplementary Files

Supplementary Data 1: Reference genome assemblies used for RNA-seq alignment. This table lists the 26 mammalian species included in the study and the specific reference genome assemblies used for aligning their RNA-seq data with HISAT2.

Supplementary Data 2: RBP and MLS-associated AS event clusters from hierarchical clustering analysis. This table provides the detailed lists of RNA-binding proteins (RBPs) and MLS-associated alternative splicing (AS) events that constitute the two main clusters (Cluster 1 and Cluster 2) identified in the hierarchical clustering analysis shown in Figure 10.

Supplementary Data 3: Comparative functional enrichment analysis of genes associated with maximum lifespan at the splicing and expression levels. This table provides a comparative functional enrichment analysis of Gene Ontology (GO) biological process terms associated with two distinct gene sets. The analysis for the MLS-AS genes was conducted using a one-sided Fisher's exact test via the Enrichr web server, with p-values adjusted for multiple comparisons using the Benjamini-Hochberg method. For the MLS-expression genes, the enrichment results are presented as reported by Lu et al.

Supplementary Data 4: Correlations between RBP motif occurrences and PSI values for MLS-associated AS events. This table lists significant Spearman correlation coefficients between the length-normalized motif frequency for a given RNA-binding protein (RBP) and the Percent Spliced-In (PSI) values for specific MLS-associated alternative splicing (AS) events, two-sided p-value, and Benjamini-Hochberg corrected false discovery rate (FDR).

Supplementary Data 5: Positively MLS-associated AS events significantly upregulated by PAPP-A inhibitor treatment. This table lists the specific positively correlated MLS-associated alternative splicing (pos-MLS AS) events that showed a significant increase in exon inclusion in mice treated with a PAPP-A inhibitor compared to controls. Significance of the increase was determined using a one-sided Fisher's exact test on median read counts, and the resulting p-values were adjusted for multiple comparisons using the Benjamini Hochberg method.
