# Supplementary material for: The implications of alternative splicing regulation for maximum lifespan
Source: Nat Commun. 2025 Nov 24;16:10317. doi: 10.1038/s41467-025-65339-1 (PMC12644568; doi:10.1038/s41467-025-65339-1)
Supplement: Supplementary file 8 — Reporting Summary [file 41467_2025_65339_MOESM8_ESM.pdf]

nature portfolio

Corresponding author(s): Liang Chen, Sika Zheng

Last updated by author(s): Oct 2, 2025

Reporting Summary

Nature Portfolio wishes to improve the reproducibility of the work that we publish. This form provides structure for consistency and transparency in reporting. For further information on Nature Portfolio policies, see our Editorial Policies and the Editorial Policy Checklist.

Statistics

For all statistical analyses, confirm that the following items are present in the figure legend, table legend, main text, or Methods section.

n/a	Confirmed	
☐	☒	The exact sample size (*n*) for each experimental group/condition, given as a discrete number and unit of measurement
☒	☐	A statement on whether measurements were taken from distinct samples or whether the same sample was measured repeatedly
☐	☒	The statistical test(s) used AND whether they are one- or two-sided<br>*Only common tests should be described solely by name; describe more complex techniques in the Methods section.*
☐	☒	A description of all covariates tested
☐	☒	A description of any assumptions or corrections, such as tests of normality and adjustment for multiple comparisons
☐	☒	A full description of the statistical parameters including central tendency (e.g. means) or other basic estimates (e.g. regression coefficient) AND variation (e.g. standard deviation) or associated estimates of uncertainty (e.g. confidence intervals)
☐	☒	For null hypothesis testing, the test statistic (e.g. *F*, *t*, *r*) with confidence intervals, effect sizes, degrees of freedom and *P* value noted<br>*Give P values as exact values whenever suitable.*
☒	☐	For Bayesian analysis, information on the choice of priors and Markov chain Monte Carlo settings
☒	☐	For hierarchical and complex designs, identification of the appropriate level for tests and full reporting of outcomes
☐	☒	Estimates of effect sizes (e.g. Cohen's *d*, Pearson's *r*), indicating how they were calculated

Our web collection on statistics for biologists contains articles on many of the points above.

Software and code

Policy information about availability of computer code

Data collection	All data used in this study was obtained from publicly available resources, and acquisition procedures can be found in the manuscript under Data acquisition section.
Data analysis	We used R 4.3.0 and Python v3.7.4 for the majority of data analysis including correlation study between MLS/age and splicing. We used quality control tool: Trim_galore v0.6.6, alignment tools: HISAT2 v2.2.1, StringTie v2.1.5,Blast v2.10.1, splicing identification tool SUPPA v2.3, Enrichr (https://maayanlab.cloud/Enrichr/), DAVID (https://davidbioinformatics.nih.gov/), and RBPmap (http://rbpmap.technion.ac.il/). The custom code is available in Zenodo under accession code https://zenodo.org/records/16042325.

For manuscripts utilizing custom algorithms or software that are central to the research but not yet described in published literature, software must be made available to editors and reviewers. We strongly encourage code deposition in a community repository (e.g. GitHub). See the Nature Portfolio guidelines for submitting code & software for further information.

Data

Policy information about availability of data

All manuscripts must include a data availability statement. This statement should provide the following information, where applicable:

- Accession codes, unique identifiers, or web links for publicly available datasets
- A description of any restrictions on data availability
- For clinical datasets or third party data, please ensure that the statement adheres to our policy

All data used in this study are publicly available. GTEx data were acquired from the Genotype Tissue Expression (GTEx) consortium (https://gtexportal.org/). Other 26 mammals data were downloaded from the GEO database (GSE181413 and GSE190756).
The custom data used and generated in this study are available in Zenodo under accession code https://zenodo.org/records/16042325. Source data are provided with this paper.

Research involving human participants, their data, or biological material

Policy information about studies with human participants or human data. See also policy information about sex, gender (identity/presentation), and sexual orientation and race, ethnicity and racism.

Reporting on sex and gender	We did not perform sex- or gender-based analysis. We consider all the samples as a whole.
Reporting on race, ethnicity, or other socially relevant groupings	Not relevant in this study.
Population characteristics	Not relevant in this study.
Recruitment	The data used in this study were from public available dataset, no new recruitment was performed.
Ethics oversight	GTEx was approved by the NIH with appropriate ethics oversight.

Note that full information on the approval of the study protocol must also be provided in the manuscript.

Field-specific reporting

Please select the one below that is the best fit for your research. If you are not sure, read the appropriate sections before making your selection.

☒ Life sciences ☐ Behavioural & social sciences ☐ Ecological, evolutionary & environmental sciences

For a reference copy of the document with all sections, see nature.com/documents/nr-reporting-summary-flat.pdf

Life sciences study design

All studies must disclose on these points even when the disclosure is negative.

Sample size	Sample sizes were defined by available public dataset (GTEx, GSE181413 GSE190756 and GSE144615)
Data exclusions	No data were excluded.
Replication	All the software and data used in this study are publicly available for the replication of the results.
Randomization	Randomization was not applicable to this observational study, as groups were defined by intrinsic sample characteristics (e.g., species, age). Confounding variables in the human dataset were controlled for by including known covariates (sex, genotyping principal components, PEER factors) in the statistical model.
Blinding	The study was purely computational, blinding was not relevant to our study.

Reporting for specific materials, systems and methods

We require information from authors about some types of materials, experimental systems and methods used in many studies. Here, indicate whether each material, system or method listed is relevant to your study. If you are not sure if a list item applies to your research, read the appropriate section before selecting a response.

Materials & experimental systems

n/a	Involved in the study
☒	☐ Antibodies
☒	☐ Eukaryotic cell lines
☒	☐ Palaeontology and archaeology
☐	☒ Animals and other organisms
☒	☐ Clinical data
☒	☐ Dual use research of concern
☒	☐ Plants

Methods

n/a	Involved in the study
☒	☐ ChIP-seq
☒	☐ Flow cytometry
☒	☐ MRI-based neuroimaging

Animals and other research organisms

Policy information about studies involving animals; ARRIVE guidelines recommended for reporting animal research, and Sex and Gender in Research

Laboratory animals	C57BL/6 mice, Norway rats, golden hamsters, capybaras, pacas, guinea pigs, chinchillas, nutria, bushy tail rats, naked mole rats, Damaraland mole rats, African spiny mice, Octodon degus, Chinese hamster, and Ellobius lutescens
Wild animals	Beavers, deer mice, muskrats, woodchucks, chipmunks, eastern mole, wild type mice, star-nosed mole, red, gray squirrels, blind mole rats, and short-tailed shrews
Reporting on sex	We did not perform sex- or gender-based analysis. We consider all the samples as a whole.
Field-collected samples	n/a
Ethics oversight	This study only used publicly available datasets obtained from the Gene Expression Omnibus (GEO) repository hosted by the National Center for Biotechnology Information (NCBI). All data used in this study were previously collected and deposited by other researchers, and these datasets are freely accessible for research purposes.

Note that full information on the approval of the study protocol must also be provided in the manuscript.

Plants

Seed stocks	n/a
Novel plant genotypes	n/a
Authentication	n/a
